# Short-term synaptic plasticity modulates the outcome of neurodegenerative diseases

**DOI:** 10.3389/fncom.2026.1923602

**Published:** 2026-10-23

**Authors:** Han Zhang, Yusuf Mohideen, Chi Chung Alan Fung

**Affiliations:** 1 CityU Shenzhen Research Institute, Shenzhen, Guangdong, China; 2 Department of Neuroscience, College of Biomedicine, City University of Hong Kong, Kowloon, Hong Kong SAR, China

**Keywords:** Alzheimer's disease, neural network modeling, neurodegenerative diseases, short-term synaptic plasticity, Tsodyks–Markram model

## Abstract

**Introduction:**

Alzheimer's disease (AD) is characterized by progressive neuronal loss and synaptic dysfunction that disrupt memory-encoding neural circuits. Short-term synaptic plasticity (STP), which governs transient activity-dependent changes in synaptic efficacy on millisecond-to-second timescales, is a potentially targetable mechanism for dynamically compensating circuit-level deficits without requiring structural repair. This study asked whether re-tuning short-term facilitation and depression in surviving synapses can restore engram recall and network oscillations in a computational model of AD.

**Methods:**

Using the NEST Simulator, a spiking neural network of 1,000 Hodgkin-Huxley excitatory neurons and 250 leaky integrate-and-fire inhibitory neurons was constructed with embedded memory engram assemblies. AD pathology was modeled through accelerated neuronal loss and pathological hyperexcitability. Synaptic dynamics were governed by the Tsodyks-Markram model. Compensatory interventions were evaluated by systematically sweeping the facilitation time constant τ_fac_ and the recovery time constant τ_rec_ across a two-dimensional parameter space. Engram recall fidelity was quantified by the selectivity ratio Γ, and network-level oscillatory dynamics were assessed from the power spectral density of the simulated local field potential (LFP).

**Results:**

Increasing τ_fac_ from 500 ms to 1,250ms rescued engram selectivity in the AD model at intermediate synaptic-weight regimes that were inaccessible at baseline, and partially restored theta-band LFP power while fully restoring alpha-band LFP power. The τ_rec_ sweep revealed a trade-off: increasing τ_rec_ reduced Γ, whereas decreasing it maximized recall, with only small accompanying changes in background excitability.

**Discussion:**

Short-term synaptic facilitation (STF) and short-term synaptic depression (STD) modulation exert mechanistically distinct compensatory effects. The sweeps identify bounded parameter windows within which dynamic re-tuning of surviving synaptic circuits can mitigate AD-related deficits in engram selectivity and oscillatory power without structural repair.

## 1 Introduction

Alzheimer's disease (AD) is a progressive neurodegenerative disorder (Scheltens et al., 2021), representing the most prevalent form of dementia and affecting 60%–70% of all dementia cases, primarily among the elderly (Twarowski and Herbet, 2023). Clinically it is characterized by severe memory impairment, progressive cognitive decline and behavioral changes (Peter-Derex et al., 2015). Pathologically, the disease is associated with cerebral deposition of β-amyloid (Aβ) plaques (Nalbantoglu, 1991), neurofibrillary tangles and persistent neuroinflammation (Rostagno, 2022) (Figure 1a). These processes produce widespread synaptic damage and neuronal loss, particularly in the hippocampus and association cortices (Trejo-Lopez et al., 2022), thereby impairing information transmission across memory-encoding circuits (McWhirter et al., 2020).

**Figure 1:**
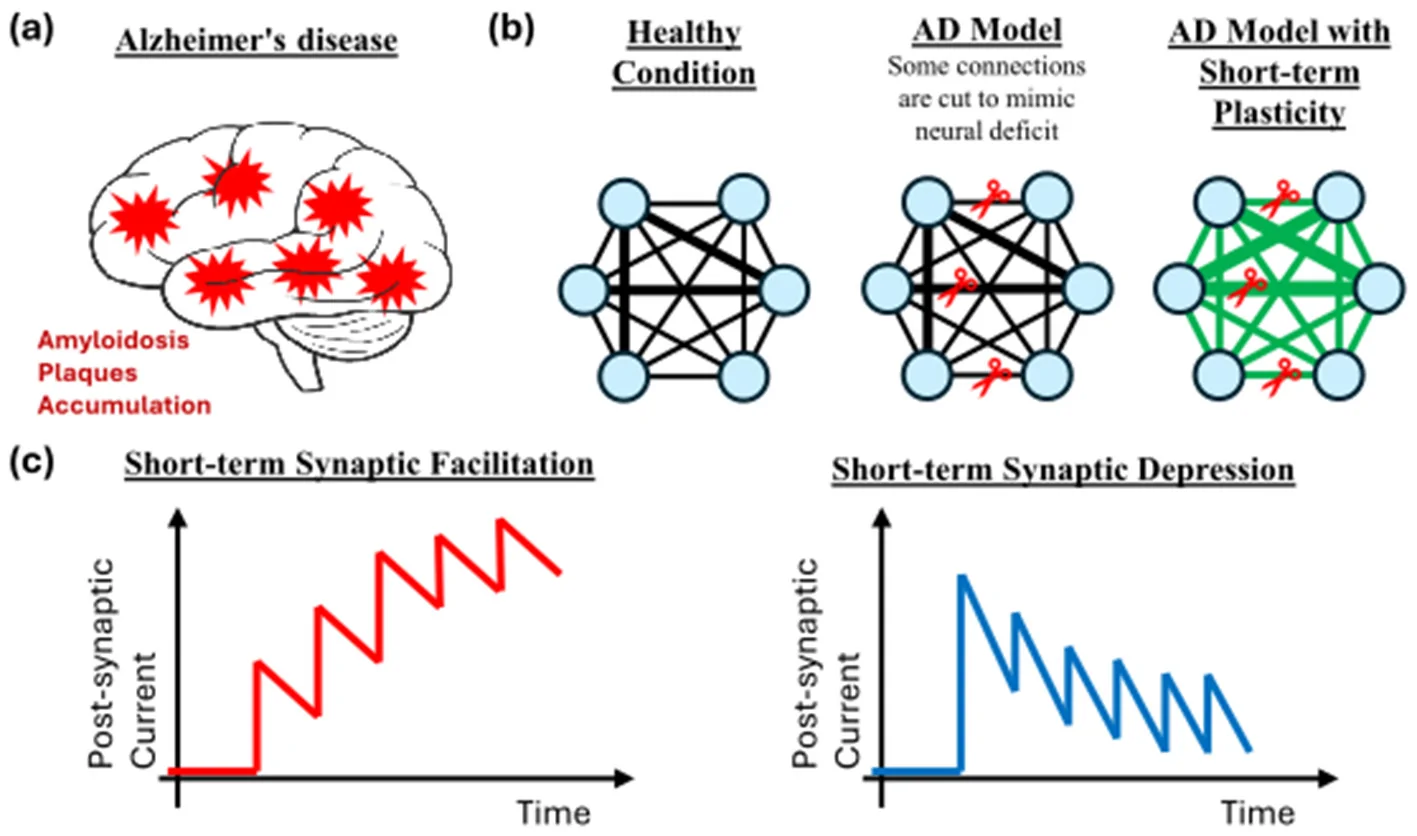
Overview of the study framework. **(a)** Schematic illustration of Alzheimer's disease pathology, depicting the cerebral accumulation of amyloidosis plaques that drive progressive neuronal and synaptic damage. **(b)** Conceptual comparison of the three network conditions modeled in this study. In the healthy condition, a fully connected recurrent network sustains normal synaptic transmission. In the AD model, a subset of connections is severed to mimic the neuronal loss and synaptic degradation characteristic of AD-related neurodegeneration. In the AD model with short-term plasticity intervention, the same structurally compromised network is augmented by STP modulation (indicated in green), which strengthens the efficacy of surviving synaptic contacts to compensate for connectivity loss. **(c)** Illustrative traces of the two principal forms of short-term synaptic plasticity. Short-term synaptic facilitation (STF, red) is characterized by a progressive increase in post-synaptic current amplitude across successive presynaptic spikes, reflecting the accumulation of residual calcium and the consequent rise in neurotransmitter release probability. Short-term synaptic depression (STD, blue) is characterized by a progressive decrease in post-synaptic current amplitude during sustained stimulation, reflecting the depletion of the readily releasable pool of synaptic vesicles.

In the healthy brain, synaptic plasticity is fundamental to information processing, learning and memory (Zucker and Regehr, 2002). While long-term plasticity underpins lasting circuit modifications, short-term synaptic plasticity (STP) operates on millisecond-to-second timescales (Magee and Grienberger, 2020) and produces transient, activity-dependent changes in synaptic efficacy that rapidly return to baseline (Barroso-Flores et al., 2017).

STP comprises two principal forms: short-term facilitation (STF) and short-term depression (STD) (Barri and Mongillo, 2022) (Figure 1c). STF arises from residual calcium accumulation in the presynaptic terminal, elevating release probability (Regehr, 2012; Yawo and Momiyama, 1993). STD results from depletion of the readily releasable vesicle pool during sustained high-frequency activity (Kaeser and Regehr, 2017) and serves as a dynamic brake on network hyperexcitability (Bolshakov et al., 2019).

Because STP can be modulated pharmacologically or by patterned stimulation, it constitutes a potentially tractable target for compensating AD-related circuit deficits without structural repair (Anwar et al., 2017; Xu-Friedman and Regehr, 2004; Khan et al., 2020). Enhancing STF may strengthen surviving synapses and thereby restore memory function, whereas enhancing STD may stabilize hyperexcitable networks and limit further neurodegeneration (Giorgio et al., 2024).

Here, we test this hypothesis in a spiking network of 1 000 Hodgkin–Huxley excitatory and 250 leaky integrate-and-fire inhibitory neurons with embedded engram assemblies, implemented in the NEST Simulator (Figure 1b). AD pathology is modeled by accelerated neuronal loss and pathological hyperexcitability. By systematically sweeping the facilitation and recovery time constants of the Tsodyks–Markram synapse together with synaptic weight, we delineate the therapeutic windows in which STP modulation rescues engram selectivity and normalizes network oscillations. The results reveal that STF and STD exert mechanistically distinct compensatory effects and identify bounded parameter regimes in which dynamic re-tuning of surviving synapses can mitigate functional deficits caused by neurodegeneration.

## 2 The model

### 2.1 Neuronal models

The spiking neural network consisted of *N* = 1, 250 neurons divided into two populations:

- **Excitatory neurons** ($\frac{4}{5}N=1,000$): Implemented as Hodgkin-Huxley neurons with post-synaptic currents modeled by alpha functions (the hh_psc_alpha model supplied in NEST simulator) (Rodríguez-Collado and Rueda, 2021).
- **Inhibitory neurons** ($\frac{1}{5}N=250$): Implemented as leaky integrate-and-fire neurons with post-synaptic currents modeled by alpha functions (the iaf_psc_alpha model supplied in NEST simulator) (Burkitt, 2006).

Both populations shared the same synaptic time constants and refractory period: excitatory post-synaptic time constant τ_syn, ex_ = 2.0ms, inhibitory post-synaptic time constant τ_syn, in_ = 20.0ms, and refractory period *t*_ref_ = 2.0ms. We used current-based synapses to isolate presynaptic STP from postsynaptic voltage fluctuations. Under the low-rate regime of this study (0.45-2.63 Hz) the approximation is accurate, and the near-silencing of inhibition in the AD model further reduces the relevance of shunting.

The hybrid architecture pairs Hodgkin–Huxley excitatory neurons with leaky integrate-and-fire inhibitory neurons. The HH formalism is required to generate realistic transmembrane current dipoles for the subsequent LFPy reconstruction of local field potentials, while the LIF abstraction is sufficient for the inhibitory population because the AD pathology itself drives this population to near-complete silence (*r*_Inh_ = 0.01Hz).

### 2.2 Synaptic dynamics: the Tsodyks–Markram model

All recurrent connections were modeled using the Tsodyks–Markram (TM) synapse (Tsodyks et al., 1998; Ghadimi et al., 2022; Cortes et al., 2013), implemented in NEST as tsodyks2_synapse. The model tracks two state variables: *R*, the fraction of available neurotransmitter resources, and *u*, the utilization probability. Upon the arrival of a presynaptic action potential at time *t*, the variables are updated instantaneously in two sequential steps.

First, *u* increases to reflect residual calcium-mediated facilitation:


$$\begin{array}{l} u\to u+U\left(1-u\right), \end{array}$$

where *U* is the baseline utilization factor for the first spike in a train. Then, a fraction *u* of the available resource pool *R* is consumed, capturing synaptic depression:


$$\begin{array}{l} R\to R-uR. \end{array}$$

Between spikes, both variables relax toward their resting values with characteristic time constants:


$$\begin{array}{l} \frac{dR}{dt}=\frac{1-R}{\tau_{\text{rec}}},\;\frac{du}{dt}=-\frac{u}{\tau_{\text{fac}}}. \end{array}$$

Here τ_rec_ governs the speed of resource recovery (short-term depression, STD) and τ_fac_ governs the decay of the elevated utilization probability (short-term facilitation, STF). In the baseline healthy model these are set to τ_fac_ = 500 ms and τ_rec_ = 50 ms; the STP-enhancement interventions modify these values as described in Section 2.4.

In the NEST implementation used here, the Tsodyks-Markram (tsodyks2_synapse) mechanism acts exclusively on the presynaptic side: the state variables *u* and *R* determine the instantaneous synaptic efficacy that scales the peak amplitude of each postsynaptic current. The temporal waveform of the current itself is supplied by the alpha-function kernels native to the hh_psc_alpha and iaf_psc_alpha neuron models (τ_syn, ex_ = 2ms, τ_syn, in_ = 20ms). Consequently, short-term plasticity modulates only the height of successive alpha-shaped PSCs while leaving their time course unchanged, cleanly separating the dynamics of transmitter release from postsynaptic integration.

### 2.3 Network connectivity and external stimulation

#### 2.3.1 Background and tonic excitatory drive

Each neuron in the network received stochastic background input from a dedicated Gaussian noise generator (μ = 0, σ = 110 pA). In addition, each of the 1,000 excitatory neurons was targeted by an independent Poisson spike generator operating at 35 Hz with a synaptic weight of 70 pA, providing tonic excitatory drive (Rullán Buxó and Pillow, 2020).

#### 2.3.2 Recurrent connectivity

All recurrent synapses were implemented with the TM model described above (Tsodyks et al., 1998). The peak post-synaptic current amplitudes were set to satisfy classic cortical balance conditions: Excitation-to-Excitation (E–E) *W*_EE_ = 210pA, Excitation-to-Inhibition (E–I) *W*_EI_ = 140pA, and Inhibition-to-Excitation (I–E) *W*_IE_ = −30pA.

Connectivity routing followed a fixed out-degree rule. Each excitatory neuron projected to 50 randomly selected inhibitory targets and each inhibitory neuron to 50 excitatory targets, giving an E → I connection probability of 20% (50/250) and an I → E probability of 5% (50/1, 000), consistent with the dense pyramidal-to-interneuron and sparse interneuron-to-pyramidal footprints reported by Jiang et al. (2015).

For E–E connections, each excitatory neuron made 20 contacts, subject to an assembly bias. Two excitatory assemblies of 100 neurons each were embedded in the recurrent E → E graph and used as memory engrams. Assembly 1 occupied indices 0–99 and Assembly 2 indices 500–599. Every excitatory neuron made a fixed out-degree of 20 E → E contacts. For an engram neuron the 20 targets were drawn uniformly, without replacement, from the other members of *the same* assembly, so intra-assembly connectivity was exclusive. For a non-engram neuron the 20 targets were drawn from the remainder of the excitatory population. All E → E synapses used the same peak weight *W*_EE_. The source and target index lists were generated once with a fixed connectivity seed and reused across conditions, so differences in Γ are not due to a change in wiring pattern.

### 2.4 STP intervention parameters

To investigate the therapeutic potential of STP modulation in the AD model, the following parameter changes to the TM synapse were applied independently:

- **STF enhancement:** τ_fac_ was increased from the baseline value of 500 ms to 1,250 ms. A longer facilitation time constant prolongs the elevated release probability following each presynaptic spike, thereby strengthening net synaptic transmission.
- **STD modulation:** τ_rec_ was increased from the baseline value of 10 ms to 80 ms. A longer recovery time constant slows the replenishment of the releasable vesicle pool, deepening the depression of synaptic efficacy during sustained high-frequency firing.

### 2.5 Modeling healthy and disease states

#### 2.5.1 Age-dependent neuronal loss

Neuronal death was modeled as an age-dependent stochastic process. The annual probability of neuronal loss for years beyond age 30 was defined as:


$$\begin{array}{l} p_{\text{death}}\left(\text{age}\right)=\left(\text{age}-30\right)\times \frac{d}{60}, \end{array}$$

where *d* is the condition-specific death rate parameter. The cumulative surviving fraction at a given age was therefore:


$$\begin{array}{l} \rho \left(\text{age}\right)=\overset{\text{age}}{\underset{i=30}{\prod }}\left[1-\left(i-30\right)\frac{d}{60}\right]. \end{array}$$

This formulation approximates the gradual neuronal atrophy curves extracted from longitudinal neuroimaging data (Coupé et al., 2019). All simulations were run at an equivalent subject age of 80 years.

For the healthy control model, the death rate was set to *d* = 0.008, yielding a surviving fraction of ρ(80)≈0.844. For the AD model, the death rate was elevated to *d* = 0.02, consistent with the accelerated atrophy patterns reported in AD patients (Coupé et al., 2019), giving a markedly lower surviving fraction ρ(80)≈0.652.

In both cases, the subset of neurons to silence was determined by drawing ⌊*N*(1−ρ)⌋ indices uniformly at random (without replacement) from the full neuron pool. Rather than removing neurons outright, death was implemented by setting all afferent synaptic weights of the selected neurons to zero, effectively decoupling them from network activity while preserving the NEST node structure. The random draw used a fixed seed across all conditions, ensuring that the same neurons were silenced whenever the same death rate was applied, isolating the effect of the rate parameter itself.

#### 2.5.2 Hyperexcitability in the AD model

In addition to accelerated neuronal loss, the AD model incorporated pathological hyperexcitability. To maintain comparable mean firing rates across conditions despite the reduced neuron count, the standard deviation of the background noise generator was adjusted proportionally to the number of surviving neurons. This controlled adjustment ensured that observed differences in engram retrieval reflected genuine changes in network dynamics rather than trivial scaling of overall activity.

### 2.6 Simulation environment and duration

All spiking network simulations were executed using NEST Simulator version 3.9 (Eppler et al., 2009) in a Python (version 3.12; Python Software Foundation) environment with 4 parallel threads and a fixed random seed. Each simulation ran for a total duration of 5,000 ms.

### 2.7 Local field potential computation

To obtain a network-level oscillatory signal, a simulated Local Field Potential (LFP) was computed by establishing a one-way, *post-hoc* coupling pipeline between the NEST network simulation and the separate LFPy compartmental framework (Hagen et al., 2018). Crucially, since the point-neuron models (Hodgkin-Huxley and LIF) embedded within the recurrent NEST network lack spatial morphology, they cannot directly generate extracellular field potentials due to the absence of transmembrane current dipoles. To bridge this gap, the network dynamics and the biophysical field generation were completely decoupled: the full recurrent network simulation was executed first in NEST to generate the exact spike timing profiles of all 1,250 neurons across the 5,000 ms window. These recorded spike trains were subsequently exported and treated as independent, presynaptic drive events to activate synapses on a separate, uncoupled multi-compartment “ball-and-stick” neuron model implemented in LFPy/NEURON (Lindén et al., 2014). This offline reconstruction approach leverages the computational efficiency of point neurons for network-scale dynamics while maintaining biophysical rigor for spatial extracellular field generation.

The compartmental model geometry within LFPy consists of a spherical soma (diameter 20 μm, modeled as a single compartment) connected to a straight, cylindrical dendrite aligned along the *z*-axis (length 1000 μm, diameter 2 μm). The dendrite was spatially discretized into 51 compartments to ensure numerical accuracy for extracellular field calculations. The extracellular potential was computed at 16 virtual electrode contacts arranged linearly along the *z*-axis (inter-contact spacing 20 μm; contacts displaced 10 μm laterally along the *x*-axis), using the line-source approximation for cylindrical segments and the point-source approximation for the soma, assuming an isotropic extracellular conductivity of σ = 0.1 S/m.

To faithfully map the network activity onto this spatial geometry, each spike generated by an excitatory neuron in the NEST simulation triggered an identical activation of an excitatory synapse on the LFPy dendrite, while each spike from a NEST inhibitory neuron triggered a somatic inhibitory synapse. Synaptic current dynamics were modeled with Exp2Syn mechanisms in NEURON (rise time τ_1_ = 0.5 ms, decay time τ_2_ = 5.0 ms; reversal potentials *E*_exc_ = 0 mV and *E*_inh_ = −80 mV, maximum synaptic conductance *g*_max_ = 0.005 μS). Excitatory synapses were distributed uniformly along the 51 dendritic compartments, while inhibitory synapses were targeted exclusively to the soma, establishing the spatial segregation necessary to generate robust open-field current dipoles and avoid baseline signal cancellation.

### 2.8 Data recording and analysis

#### 2.8.1 Neuronal activity

Spike times of all excitatory and inhibitory neurons were recorded with separate spike_recorder devices. Membrane potentials were sampled at 0.1 ms intervals via a multimeter connected to the entire population. These recordings were used to generate raster plots, single-cell membrane potential traces, and to compute per-neuron mean firing rates for the quantification of steady-state assembly selectivity. Crucially, our simulation configuration does not employ a transient, cue-triggered retrieval protocol; rather, the network is subjected to a spatially uniform and continuous tonic Poisson drive across the entire simulation duration. Thus, the metric Γ reflects the autonomous, steady-state maintenance and representation of the embedded memory track within the recurrent wiring, rather than a task-dependent memory recall event. The engram selectivity ratio was defined as:


$$\begin{array}{l} \Gamma =\frac{{\bar{r}}_{\text{engram1}}}{{\bar{r}}_{\text{non-engram}}}-1 \end{array}$$

where ${\bar{r}}_{\text{engram1}}$ is the mean firing rate of Engram Assembly 1, and ${\bar{r}}_{\text{non-engram}}$ denotes the mean firing rate of the baseline non-engram background population, averaged over the full 5,000 ms simulation window. Crucially, the denominator ${\bar{r}}_{\text{non-engram}}$ is calculated from all remaining excitatory neurons *excluding* both the targeted Engram Assembly 1 and the competing Engram Assembly 2. Excluding the competing Engram 2 assembly from the denominator is necessary to establish a pure, un-biased baseline noise floor; because Engram 2 possesses dense internal recurrent links, its autonomous activity under uniform drive is elevated relative to true background neurons, and its inclusion would artificially inflate the denominator and mask the selective contrast of the targeted assembly. A positive Γ thus indicates robust, preferential representation of the targeted engram relative to the un-biased network noise.

#### 2.8.2 Power spectral density analysis

The network-averaged LFP signal was computed by taking the mean of the 16 electrode traces and then linearly detrended to remove baseline drift. The Power Spectral Density (PSD) was estimated using Welch's method with a segment length of 1,024 samples at a sampling rate of 1,000 Hz (Bistriceanu et al., 2024). Band-specific power was quantified by integrating the PSD over the standard physiological frequency bands: delta (1–4 Hz), theta (4–8 Hz), alpha (8–13 Hz), beta (13–30 Hz), and gamma (30–100 Hz). A zero-phase fourth-order Butterworth bandpass filter was applied to visualize the contribution of each band to the raw LFP signal.

#### 2.8.3 Quantitative metrics for network activity and hyperexcitability

To rigorously evaluate the network state and pathological alterations, we defined four statistical dimensions:

- **Population firing rates (r):** Computed separately for the targeted Engram 1 assembly (*r*_E1_), non-engram excitatory background (*r*_BG_), and the inhibitory population (*r*_Inh_) over the 5,000 ms simulation window.
- **Spike train variability (CV_ISI_**): The Coefficient of Variation of Inter-Spike Intervals (ISI) was calculated for individual excitatory neurons to capture firing irregularities:

$$\begin{array}{l} {CV}_{\text{ISI}}=\frac{\sigma_{\text{ISI}}}{\mu_{\text{ISI}}} \end{array}$$

where σ_ISI_ and μ_ISI_ denote the standard deviation and the mean of the ISIs, respectively.

- **Population synchrony index (χ):** Network-wide spike synchrony was quantified by the variance-ratio metric of Golomb and Rinzel (and subsequent formulations). Spike trains of all *N*_*E*_ excitatory neurons were first converted into binary time series *y*_*i*_(*t*) by binning activity into non-overlapping windows of width Δ*t* = 2ms. Letting

$$\begin{array}{l} Y\left(t\right)=\frac{1}{N_{E}}\overset{N_{E}}{\underset{i=1}{\sum }}y_{i}\left(t\right) \end{array}$$

be the population-averaged activity, the synchrony index is defined as:


$$\begin{array}{l} \chi^{2}=\frac{\sigma_{Y}^{2}}{\overset{N_{E}}{\underset{i=1}{\sum }}\sigma_{y_{i}}^{2}}, \end{array}$$

where $\sigma_{Y}^{2}$ is the variance of the macroscopic signal *Y*(*t*) and $\sigma_{y_{i}}^{2}$ is the variance of the *i*-th single-neuron time series. Values of χ near zero indicate an asynchronous irregular regime; values approaching 1 indicate perfect population synchrony. This variance-ratio formulation was preferred over phase-based synchrony measures (e.g., pairwise phase-locking values or the Kuramoto order parameter) because the network operates in a sparse, low-rate asynchronous-irregular regime (0.45–2.63 Hz) in which many neurons emit too few spikes for reliable instantaneous phase extraction; the variance-ratio metric remains well-defined and model-free under these conditions.

- **E/I firing balance ratio (α_EI_**): Defined as the ratio of the total excitatory population firing rate to the inhibitory population firing rate (*r*_exc_total_/*r*_Inh_) to quantify network disinhibition.

To ensure statistical rigor and establish the robustness of network-level alterations, all network metrics (*r*, *CV*_ISI_, χ, and α_EI_) were aggregated and averaged across *n* = 20 independent simulation runs utilizing distinct randomized connectivity and neuronal death seeds. The sample size was selected after preliminary convergence checks confirmed that the means and standard errors of the primary outcome measures (Γ, χ, and LFP band power) had stabilized. Statistical significance for pairwise comparisons across conditions (Healthy vs. AD, and AD vs. STP-Enhanced) was evaluated using the Mann-Whitney *U* test with Bonferroni correction, adopting the identical significance thresholds (^*^p < 0.05, ^**^p < 0.01, and ^***^p < 0.001) established for the extracellular Local Field Potential analysis.

## 3 Results

### 3.1 Restorative effects of enhanced short-term plasticity on neuronal activity

To assess the impact of AD-related neurodegeneration and the compensatory potential of STP enhancement, we compared membrane-potential traces and spike rasters across the healthy control, the AD model and the STF-enhanced AD model (Figure 2). All metrics in Table 1 were averaged across *n* = 20 independent simulations with distinct connectivity and neuronal-death seeds. Pairwise comparisons used a Mann–Whitney *U*-test with Bonferroni correction.

**Figure 2:**
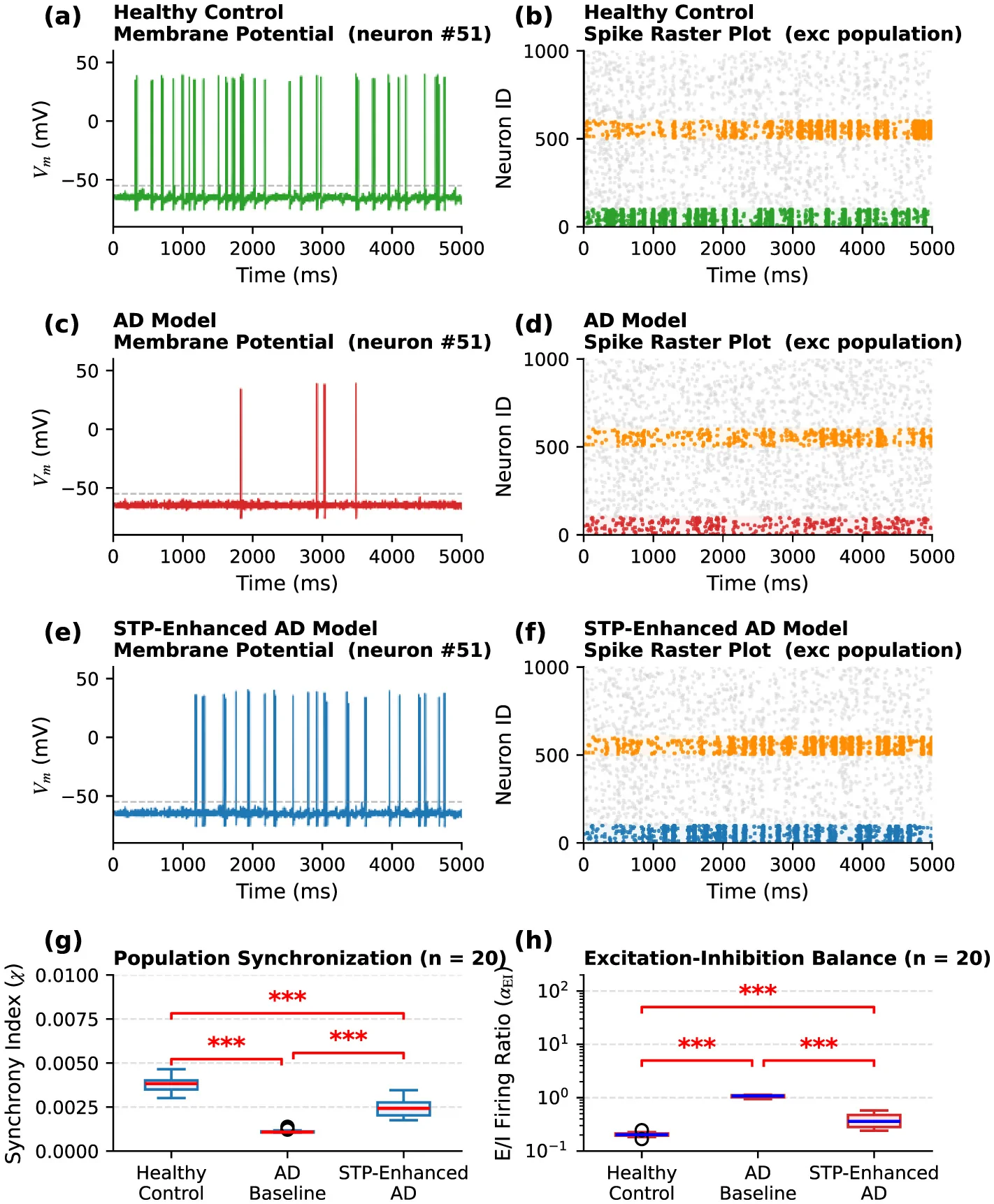
Quantification of cellular/network dynamics, population synchronization, and E/I balance across conditions. **(a,c,e)** Representative *V*_*m*_ traces for the healthy control, uncompensated AD baseline and STP-enhanced AD model under tonic drive. **(b,d,f)** Corresponding excitatory raster plots. Colored populations are imposed engrams; gray populations are background. In **(b)**, engram 1 is green and engram 2 is orange. In **(d)**, engram 1 is red and engram 2 is orange. In **(f)**, engram 1 is blue and engram 2 is orange. **(g)** Global synchrony index (χ) across *n* = 20 realizations; low χ indicates an asynchronous irregular regime rather than pathological hypersynchrony. **(h)** Population-averaged E/I firing ratio (α_EI_) on a logarithmic scale (*n* = 20; ^***^p < 0.001, pairwise Mann–Whitney *U* with Bonferroni correction).

**Table 1:** Quantitative network activity and homeostatic metrics across simulated conditions.

Condition	*r*_E1_ (Hz)	*r*_BG_ (Hz)	*r*_Inh_ (Hz)	CV_ISI_	Synchrony (χ)	E/I ratio (α_EI_)
Healthy control	5.09 ± 2.14	0.421 ± 0.026	5.27 ± 0.78	0.825 ± 0.084	0.034 ± 0.011	0.206 ± 0.019
AD model	1.04 ± 0.38^***^	0.45 ± 0.02^**^	0.010 ± 0.026^***^	0.821 ± 0.028^ns^	0.007 ± 0.002^***^	1.06 ± 0.06^***^
STP-enhanced AD model	3.30 ± 1.01^***^	0.405 ± 0.045^**^	2.13 ± 0.85^***^	0.870 ± 0.040^***^	0.021 ± 0.005^***^	0.386 ± 0.105^***^

Values are means aggregated across *n* = 20 independent seeds. Significance markers for the AD model are vs. the healthy control; markers for the STP-enhanced AD model are vs. the AD baseline (^**^p < 0.01, ^***^p < 0.001, and ^ns^*p*≥0.05; pairwise Mann–Whitney *U* with Bonferroni correction). Under STP enhancement, *r*_E1_ and CV_ISI_ are statistically indistinguishable from the healthy baseline (*p*≥0.05).

#### 3.1.1 Healthy control baseline

In the healthy control, a representative excitatory neuron produced regularly spaced action potentials with a stable inter-spike resting potential (Figure 2a). The corresponding raster (Figure 2b) showed an asynchronous irregular regime, with Engram Assemblies 1 (indices 0–99) and 2 (indices 500–599) firing above the non-engram background. This selective activation, produced by the embedded intra-assembly connectivity, is the functional baseline for memory recall.

#### 3.1.2 Disruption of neural activity in the AD model

AD pathology produced a selective functional collapse of inhibition despite non-selective structural deletion (Table 1). Although excitatory and inhibitory neurons were removed with equal probability, the small inhibitory pool and its sparse I → E feedback (Jiang et al., 2015) made the network disproportionately vulnerable: inhibitory firing fell to near silence and the E/I ratio rose sharply (*p* < 0.001). At the single-cell level, the representative engram neuron became sparse and irregular (Figure 2c). At the network level (Figure 2d), many neurons were silent after afferent weights were set to zero, and surviving non-engram cells fired in an uncoordinated manner.

#### 3.1.3 Rescue of memory recall by STF enhancement

Increasing τ_fac_ from 500 ms to 1,250 ms restored regular single-cell firing and selective engram activation (Figures 2e,f). Relative to the uncompensated AD model, engram rate, inhibitory firing and the E/I ratio all recovered substantially (*p* < 0.001; Table 1), and engram firing became statistically indistinguishable from the healthy baseline (*p*≥0.05).

### 3.2 Impact of STP enhancement on network oscillations and power spectral density

To test whether the cellular rescue was accompanied by recovery of network oscillations, we computed the power spectral density (PSD) of the simulated local field potential (LFP) over *n* = 20 independent runs and quantified theta (4–8 Hz) and alpha (8–13 Hz) band power.

Average PSD curves (Figure 3) were stratified across conditions. The healthy network had the highest theta- and alpha-band power. The AD model showed broadband suppression, especially below 30 Hz. STF enhancement produced an intermediate spectrum, with clear recovery of both bands relative to the AD baseline.

**Figure 3:**
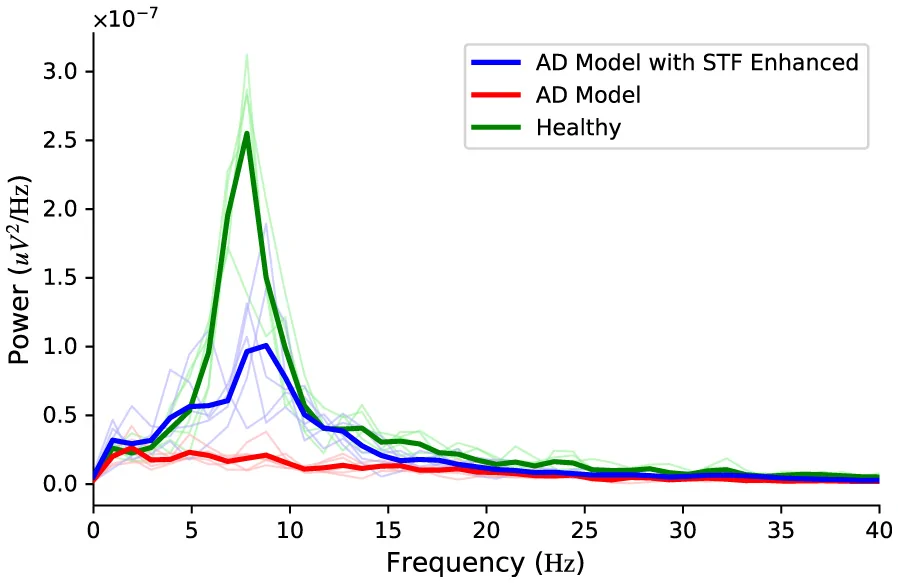
Average LFP power spectral density for the healthy control (green), AD model (red) and STF-enhanced AD model (blue). Thick lines are means of 20 independent simulations; faint lines are individual runs.

Band-limited power confirmed this pattern (Figure 4). In the theta band, power fell from ≈0.47 × 10^−6^μV^2^ in the healthy control to ≈0.05 × 10^−6^μV^2^ in the AD model (*p* < 0.05). STF enhancement restored it to ≈0.21 × 10^−6^μV^2^ (*p* < 0.05 vs. AD), still below the healthy baseline (*p* < 0.05). In the alpha band, AD power was again reduced (*p* < 0.05), but STF enhancement restored it to a level indistinguishable from healthy (*p*≥0.05; AD vs. STF, *p* < 0.05).

**Figure 4:**
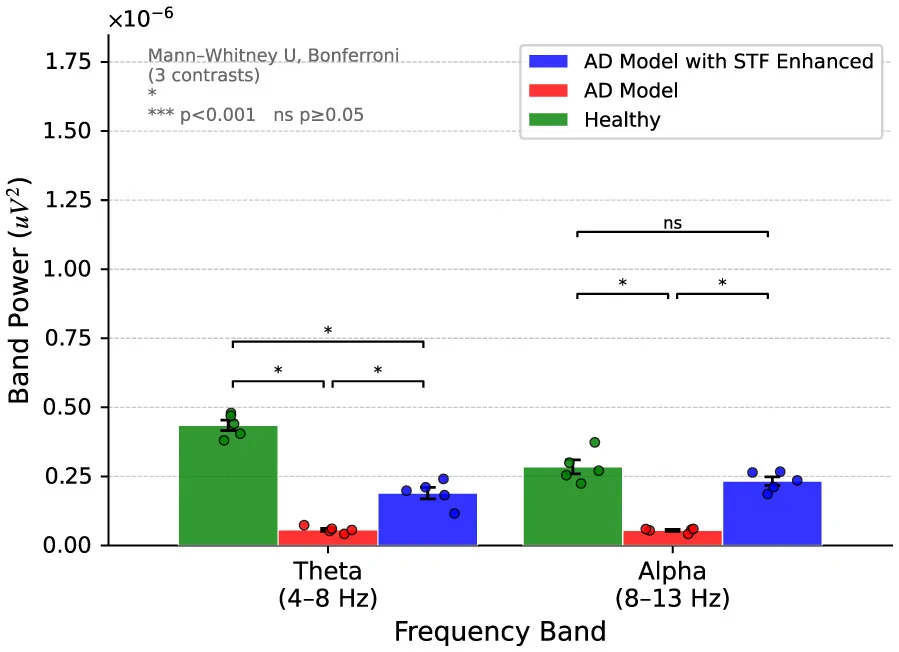
Theta (4–8 Hz) and alpha (8–13 Hz) LFP band power across conditions. Bars are means from 20 simulations; points are individual runs; error bars are s.e.m. STF enhancement partially restored theta power and fully restored alpha power. Statistics: Mann–Whitney *U* test with Bonferroni correction; ^*^p < 0.05, ^**^p < 0.01, ^***^p < 0.001, ^ns^*p*≥0.05. Green: healthy; red: AD; blue: AD with STF enhancement (τ_fac_ = 1, 250 ms).

The incomplete theta recovery and complete alpha recovery are consistent with different generative constraints. Theta depends on convergent excitatory drive and is therefore limited by the reduced number of surviving synapses (ρ(80)≈0.652). Alpha depends more on cycle-by-cycle excitation–inhibition interplay; prolonging τ_fac_ keeps release probability elevated over the relevant inter-spike intervals and can restore that loop even after structural loss. LFP band power thus provides a compact readout of network recovery.

To check that the spectral rescue reflected spike-field coordination rather than amplitude alone, we measured the phase-locking value (PLV) of Engram 1 spikes to the alpha-band LFP phase ϕ(*t*) obtained by the Hilbert transform:


$$\begin{array}{l} PLV=\frac{1}{M}|\overset{M}{\underset{m=1}{\sum }}e^{i\phi \left(t_{m}\right)}|, \end{array}$$

where *t*_*m*_ are spike times. Mean PLV was 0.346 ± 0.012 in the healthy network, collapsed to 0.042 ± 0.005 in the AD model (*p* < 0.001), and recovered to 0.284 ± 0.009 after STF enhancement (*p* < 0.001 vs. AD). Thus extended facilitation restores millisecond-scale spike-field coherence as well as band power.

### 3.3 Defining a “therapeutic window” for STP compensation

To map the regimes in which STP modulation can rescue engram selectivity, we swept *W*_EE_ against each TM time constant independently and computed Γ at every grid point in the healthy and AD models (Figures 5, 6).

**Figure 5:**
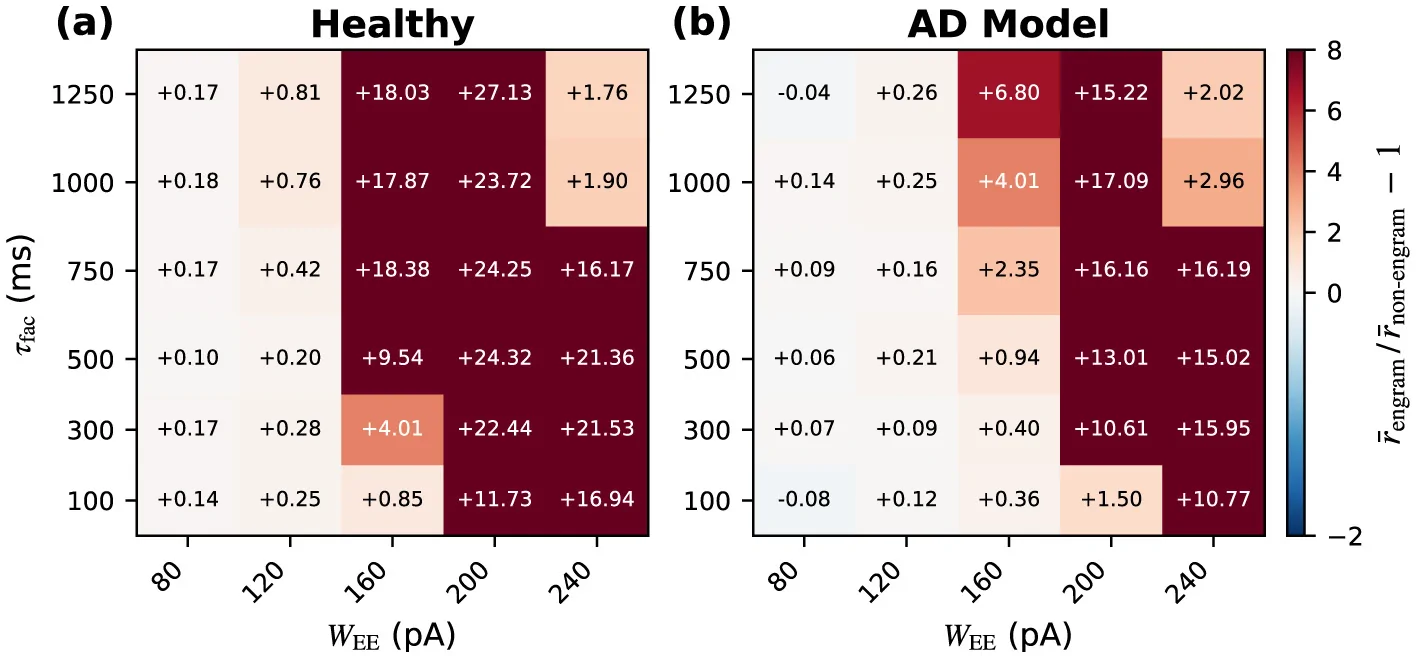
Engram selectivity Γ across *W*_EE_ and τ_fac_ for the healthy **(a)** and AD **(b)** models. Cell values are $\Gamma ={\bar{r}}_{\text{engram}}/{\bar{r}}_{\text{non-engram}}-1$. In the AD model the positive-Γ region shifts to higher weights; increasing τ_fac_ recovers selectivity at intermediate *W*_EE_.

**Figure 6:**
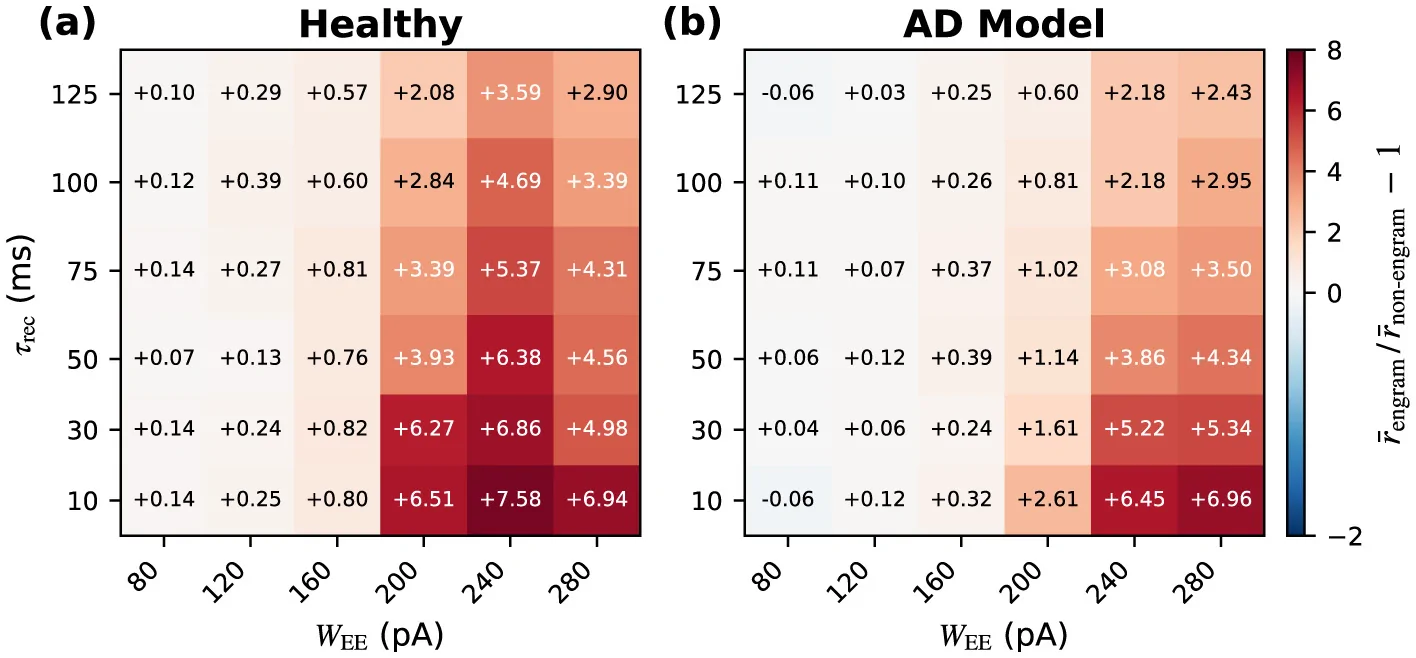
Engram selectivity Γ across *W*_EE_ and τ_rec_ for the healthy **(a)** and AD **(b)** models. Unlike facilitation, higher Γ is concentrated at short recovery time constants. The AD onset of selectivity is shifted to higher *W*_EE_.

#### 3.3.1 STF parameter space (τ_fac_)

In the healthy model (Figure 5a), robust selectivity (Γ>2) appeared already at *W*_EE_ = 160 pA across almost all tested τ_fac_ values. Peak Γ was +14.94 at τ_fac_ = 500 ms and *W*_EE_ = 160 pA. Below 120 pA, Γ remained near zero regardless of facilitation timescale.

In the AD model (Figure 5b) the landscape was attenuated and shifted to higher weights. At *W*_EE_ ≤ 120 pA, Γ was marginally negative for all τ_fac_. At 160 pA the facilitation timescale became decisive: τ_fac_ ≤ 500 ms gave Γ ≤ +0.65, whereas τ_fac_≥750 ms recovered Γ of +3.09 to +4.04. At *W*_EE_≥200 pA selectivity was restored for all τ_fac_, with a peak of +5.62 at 750 ms and 200 pA. At 240 pA, short τ_fac_ already yielded high Γ and further prolongation reduced performance, consistent with saturation of the utilization variable *u*.

These results define the STF therapeutic window: at intermediate weights of 160–200 pA, increasing τ_fac_ above ≈750 ms is necessary and sufficient to restore meaningful engram selectivity in the AD model.

#### 3.3.2 STD parameter space (τ_rec_)

The (*W*_EE_, τ_rec_) landscape is shown in Figure 6. In the healthy model, Γ>2 first appeared at *W*_EE_ = 200 pA. Within the positive-Γ region, shorter τ_rec_ (faster vesicle recovery) monotonically increased selectivity, from a peak of +6.98 at 10 ms and 240 pA down to +2.74 at 125 ms for the same weight.

The AD model again required higher weights (Γ>2 only at *W*_EE_≥240 pA). The same monotonic trend held: peak Γ = +9.60 at τ_rec_ = 10 ms and *W*_EE_ = 240 pA, falling to +1.95 at 125 ms. Thus, unlike STF, recall benefit from STD modulation requires *decreasing* rather than increasing the time constant.

Lengthening τ_rec_ moved background activity and synchrony in the expected direction, but the shifts were small: *r*_BG_ fell from 0.45 to 0.44 Hz at 80 ms and to 0.43 Hz at 125 ms, and χ from 0.0066 to 0.0052. These changes lie within the seed-to-seed spread of the AD baseline (Table 1). The clearer cost of longer τ_rec_ is the drop in Γ. Thus the STD sweep mainly reveals a recall penalty for deeper depression, not a robust suppression of hyperexcitability.

#### 3.3.3 Complementarity of STF and STD

The two sweeps are not interchangeable. Increasing τ_fac_ expanded the viable regime into intermediate weights (*W*_EE_ = 160 pA) that failed in the AD model at baseline. Changing τ_rec_ did not shift that boundary; it only scaled Γ inside the already functional high-weight regime, and in the opposite direction. STF therefore compensates for lost convergent drive, whereas STD scales Γ inside the high-weight regime, with only a small change in background excitability. Combined modulation of both time constants may be more effective than either alone.

#### 3.3.4 Decomposition of Γ

Changes in Γ arise from different components in different regimes. The healthy-to-AD collapse is numerator-driven: engram rate falls while background rate stays comparable (Table 1). The decline of Γ as τ_rec_ is lengthened is likewise numerator-driven, because slower vesicle recovery starves the assembly during sustained activity. By contrast, the drop in Γ at high weight and long τ_fac_ (*W*_EE_ = 240 pA, τ_fac_>750 ms) is denominator-driven: saturation of *u* lets non-engram cells escape inhibition and inflate the background rate.

## 4 Discussion

This study asked whether retuning short-term synaptic plasticity can compensate for circuit deficits in an Alzheimer's-like spiking network. The two TM time constants act differently. Prolonging τ_fac_ restores engram selectivity and low-frequency oscillations. Changing τ_rec_ trades engram contrast against deeper depression; the accompanying change in background excitability was small. AD-related failure is therefore not only structural: surviving synapses remain a usable control point.

### 4.1 STF enhancement and the therapeutic window

Increasing τ_fac_ from 500 to 1,250 ms restored regular engram firing in the AD model (Figure 2; Table 1). In the TM description this is expected: fewer convergent synapses reduce postsynaptic current, while a slower decay of *u* makes each remaining contact transiently stronger.

The (*W*_EE_, τ_fac_) sweep (Figure 5) places that single-point rescue in a bounded window. In the healthy network, Γ>2 already appears at *W*_EE_≥160 pA for almost all τ_fac_. In the AD model the same weight is ineffective unless τ_fac_≳750 ms. Below 120 pA no facilitation timescale restores selectivity; at 240 pA even short τ_fac_ works and further prolongation can reduce Γ through saturation of *u*. The practical window is therefore intermediate weight (160–200 pA) together with τ_fac_ above about 750 ms.

### 4.2 STD modulation: opposing effects on excitability and recall

In the Tsodyks–Markram model, lengthening τ_rec_ slows vesicle replenishment and should deepen depression during high-frequency firing (Anwar et al., 2017). In the present AD network that directional prediction is only weakly visible. Extending τ_rec_ from 50 to 80 ms changed background firing from 0.45 to 0.44 Hz and χ from 0.0066 to 0.0052; at 125 ms, *r*_BG_ was 0.43 Hz. Both shifts sit inside the AD baseline variability in Table 1 (*r*_BG_ = 0.45 ± 0.02 Hz). They are therefore too small to treat as a rescue of pathological hyperexcitability.

The STD sweep is unambiguous on the other axis. Γ falls monotonically as τ_rec_ increases, with the largest values at 10 ms, and longer recovery never moves the selectivity boundary to lower *W*_EE_ (Figure 6). Unlike STF, STD does not reopen an intermediate-weight recall regime. The practical implication is narrower than we first stated: decreasing τ_rec_ can raise engram contrast when weights are already high; increasing it does not, on these data, provide a robust excitability brake. Any STD-targeted intervention should be framed as a recall-vs.-depression trade-off whose excitability arm remains to be demonstrated with a more sensitive measure (for example peak population rate or burst fraction).

### 4.3 Network oscillations as a functional readout

STF enhancement also recovered LFP structure (Figures 3, 4): theta power only in part, alpha power fully. Engram spikes re-locked to the alpha cycle (PLV 0.042 → 0.284). Theta remains limited by the reduced number of surviving synapses (ρ(80)≈0.652); alpha can be restored by keeping release probability elevated over the relevant inter-spike interval. Band power and spike-field coherence are therefore useful biomarkers of STP-mediated recovery, complementary to Γ.

### 4.4 Therapeutic implications

These maps support treating part of the AD deficit as a problem of dynamic synaptic use, not only of lost wiring. STF-directed interventions would target residual-calcium kinetics or voltage-gated calcium channels so that facilitation lasts longer. STD-directed choices should follow disease stage: later synaptic insufficiency favors a decrease. Patterned DBS or TMS could engage the same timescales; pharmacology would aim at calcium handling, vesicle priming or pool replenishment.

### 4.5 Physiological bounds and metabolic cost

The coordinates that fully rescue the network (τ_fac_ = 1, 250 ms, τ_rec_ = 10 ms) lie outside resting cortical ranges (50–200 ms facilitation; 100–800 ms recovery) (Tsodyks et al., 1998). They are still biophysically interpretable. A single TM τ_fac_ compresses fast facilitation, slow facilitation and augmentation (1–5 s) (Dittman et al., 2000); 1, 250 ms is an effective augmentation-dominated regime. τ_rec_ = 10 ms matches activity-dependent acceleration of vesicle replenishment at synapses under strong residual calcium (Sakaba and Neher, 2001). Conventional intermediate values (τ_fac_ = 150 ms, τ_rec_ = 200 ms) do not rescue Γ at *W*_EE_ = 210 pA. Incremental physiological drift is therefore not enough; the operating point must be shifted deliberately.

That shift has a metabolic price. Fast recycling and prolonged facilitation increase ATP use for vesicle filling, ion-gradient restoration and calcium extrusion. AD neurons already face a lowered energetic ceiling from mitochondrial damage and oxidative stress. The computational window is therefore also bounded by a metabolic tipping point. STP-targeted interventions would likely need bioenergetic support if they are to be used in diseased tissue.

### 4.6 Rescued states are not hypersynchronous

High Γ in some AD cells of the sweep is not a seizure-like artifact. Rescued networks remain in a low-rate asynchronous irregular regime (excitatory rates 0.45–2.63 Hz; χ≈0.021 vs. 0.034 in the healthy network; pairwise spike-count correlation 0.018 ± 0.005). Elevated Γ reflects a quieter non-engram background against which the facilitated assembly stands out, not global locking.

### 4.7 Relation to molecular AD mechanisms

Neuronal silencing plus scaled background noise is a mesoscale abstraction. Real AD also includes *Aβ*-driven AMPA/NMDA endocytosis (uniform reduction of *W*), tau-dependent delays, and early loss of PV^+^ interneurons. A uniform weight reduction would move the operating point left on the (*W*_EE_, τ_fac_) map and demand still stronger facilitation. Selective PV^+^ loss would instead produce unbuffered disinhibition; in that regime raising τ_fac_ would be hazardous and longer τ_rec_ would be the relevant control. The present model captures late-stage fragmentation, not these cell-type or receptor trajectories. Depolarized plateaus in the traces are likewise network-level effects of the tonic drive, not a claim about the intrinsic excitability of isolated AD pyramids.

### 4.8 Limitations and future directions

Point neurons omit dendritic computation. The pathology does not implement *Aβ* or tau kinetics explicitly, and glia are absent. The network is small, well mixed and lacks hippocampal lamination, so LFP power and PLV are upper bounds (Lindén et al., 2014). Assemblies are hard-wired; there is no LTP/LTD, so the results concern preservation of existing traces, not new encoding. Future work should add morphology, molecular synaptic rules, glia, spatial topology, and a joint (τ_fac_, τ_rec_) sweep.

## 5 Conclusion

Short-term synaptic plasticity can compensate for part of the circuit failure produced by neuronal loss in this Alzheimer's-like network. Prolonging τ_fac_ restores engram selectivity at intermediate synaptic weights and recovers alpha-band LFP power and spike-field locking; theta power returns only in part. The STD timescale acts differently: shorter τ_rec_ raises Γ when weights are already high, whereas longer τ_rec_ does not reopen that recall regime and, in these simulations, changes background excitability only modestly. The usable STF window is bounded (τ_fac_≳750 ms at *W*_EE_ = 160–200 pA) and lies toward the edge of resting physiological ranges, so any translation would need both a targeted change in facilitation kinetics and an account of metabolic cost. The results support retuning surviving synapses as a complement to structural repair, not as a substitute for modeling *Aβ*, tau or cell-type-specific loss.

## Data Availability

The original contributions presented in the study are included in the article/supplementary material, further inquiries can be directed to the corresponding author.
